# A Centre Shot with Sulphur

**Published:** 1888-12

**Authors:** 


### A CENTRE-SHOT WITH SULPHUR$^{10,000}$.

Mrs. L., a lady in the forties, a native of South Carolina, has been an invalid for the last ten years, and treated here and in Europe by the best dermatologists for a rebellious eczema on her body, especially on the bends of joints, itching terribly, robbing her of sleep, taking away all appetite, and making life a burden. From childhood up she suffered from constipation, palliated off and on by the use of purgatives which still more weakened her, and she does not recollect that she ever perspired. Her courses, which always were scanty, ceased at a very early date. During the unpleasantness which the United States had with its Southern children she suffered many privations, and since then rheumatic attacks were added to the eczema, so that she suffers agony in damp weather, and, becoming a confirmed invalid, a better climate was recommended, and the family removed to California, residing at first at Stockton, and as she suffered there from intermittents, they changed to the healthier climate of Northern California. Here I made her acquaintance, and found a dried-up old woman with a broken-down constitution. For years she had on her left leg an open ulcer to which Zinc ointment was constantly applied, and still the same burning pains day and night, with excoriating discharge and sensitiveness to the least touch. Her whole skin feels subjectively hot, not objectively. The application of water burns her, and she cannot bear the touch of flannel on the eczematous spots spread out here and there over her body. Constant thirst for water, which disagrees; mouth and fauces are constantly dry and feel like cotton in it; worse in morning; all sorts of berries distress her; food oppresses her and lies like a load in stomach, though, as her husband says, she does not take enough to feed a bird. No inflammatory symptoms on eczema; skin looks rather dry; cracks easily; sometimes little vesicles, like kernels, spring up, but they soon dry in again; insomnia.

She received one powder of Sulphur$^{10,000}$, and pellets No. 30, with the advice to take two pellets night and morning. In two weeks after taking that one dose the œdema in her legs disappeared, and the ulcer began to heal. All salves being left off, she only applied some soft linen soaked in a very weak solution of Bicarbonate of Soda, and she was astonished when she could bear the water; the eczema also showed signs of disappearance; sleep and appetite returned, and she began again to look her age and feel hopeful, where before despondency ruled. About three months later the eczema was nearly gone, but a crop of suppurating boils appeared, especially in glandular regions. It may have been a mistake, but she received another dose of that Sulphur$^{10,000}$ (made by Deschere's bottle-washing machine), and is now a well woman, thanks to Homœopathy true and simple.

